# The baseline levels and risk factors for high-sensitive C-reactive protein in Chinese healthy population

**DOI:** 10.1186/s12979-018-0126-7

**Published:** 2018-09-08

**Authors:** Ying Tang, Peifen Liang, Junzhe Chen, Sha Fu, Bo Liu, Min Feng, Baojuan Lin, Ben Lee, Anping Xu, Hui Y. Lan

**Affiliations:** 1 Department of Nephrology, Sun Yat-sen Memorial Hospital, Sun Yat-sen University, Guangzhou, China; 2 Guangdong Provincial Key Laboratory of Malignant Tumor Epigenetics and Gene Regulation, Sun Yat-sen Memorial Hospital, Sun Yat-sen University, Guangzhou, China; 3 Department of Medicine and Therapeutics, Li KaShing Institute of Health Sciences, the Chinese University of Hong Kong, Hong Kong, SAR China; 4 Lui Che Woo Institute of Innovative Medicine, the Chinese University of Hong Kong, Hong Kong, SAR China; 5 Guangzhou Deling Software Technology Co., Ltd, Guangzhou, China

**Keywords:** Hs-CRP, Ageing, Inflammation, Risk factor

## Abstract

**Background:**

Recent studies show that C-reactive protein (CRP) is not only a biomarker but also a pathogenic mediator contributing to the development of inflammation and ageing-related diseases. However, serum levels of CRP in the healthy ageing population remained unclear, which was investigated in the present study.

**Methods:**

Serum levels of high sensitive C-reactive protein (hs-CRP), glucose (Glu), triglyceride (TG), cholesterol (CHOL), high-density lipoprotein cholesterol (HDL-c), low-density lipoprotein cholesterol (LDL-c), superoxide dismutase (SOD), serum creatinine (SCr), serum uric acid (SUA) were measured in 6060healthy subjects (3672 male and 2388 female, mean age:45.9 years) who received routine physical examination at Sun Yat-sen Memorial Hospital, Guangzhou, China.

**Results:**

In total of 6060 healthy people, serum levels of hs-CRP were significantly increased with ageing (*P* < 0.05), particularly in those with age over 45-year-old (1.31[0.69–2.75] vs 1.05[0.53–2.16]mg/L, *P* < 0.001). Interestingly, levels of serum hs-CRP were significantly higher in male than female population (1.24[0.65–2.57] vs 1.07[0.53–2.29]mg/L, *P* < 0.001). Correlation analysis also revealed that serum levels of hs-CRP positively correlated with age and SUA, but inversely correlated with serum levels of HDL-c and SOD (all *P* < 0.05).

**Conclusions:**

Baseline levels of serum hs-CRP are increased with ageing and are significantly higher in male than female healthy population. In addition, elevated serum levels of hs-CRP are also associated with increased SUA but decreased HDL-c and SOD. Thus, serum levels of hs-CRP may be an indicator associated with ageing in healthy Chinese population.

**Electronic supplementary material:**

The online version of this article (10.1186/s12979-018-0126-7) contains supplementary material, which is available to authorized users.

## Background

Ageing is a natural phenomenon characterized by gradual deterioration of the body structure and function with important social, public health, and economic implications. Increasing evidence shows that inflammation is one of the key mechanisms associated with many ageing-related diseases, including cancer, atherosclerosis, hypertension, diabetes mellitus, ischemic heart disease, cirrhosis, Alzheimer’s disease and other dementias and chronic diseases [1, 2].Therefore, disentangling age-related low grade inflammation may slow or delay ageing process [2]. Many inflammatory effector molecules and biomarkers, especially C-reactive protein (CRP), interleukin-6 (IL-6) and tumor necrosis factor-alpha (TNF-α), have been considered as important factors associated with ageing, ageing-related diseases or disability [3]. CRP, an acute phase protein, is primarily produced and secreted by the liver in response to IL-6.Serum levels of CRP can be measured by a high sensitivity immunoturbidometric assay termed high-sensitive CRP (hs-CRP). Many studies have shown that elevated levels of serum hs-CRP are associated with ageing and ageing-related diseases including cardiovascular disease (CVD), hypertension, diabetes mellitus, and kidney disorders [4–8].However, there is still a lack of evidence for the relationship between the baseline levels of hs-CRP and the ageing progress in general healthy population, which was investigated in this study. In addition, although many inflammatory mediators including dyslipidemia, insulin resistance, oxidative stress, and serum uric acid (SUA) have also been shown to contribute to high levels of serum hs-CRP under various disease conditions [9–16]. The link between hs-CRP and these factors in the healthy population remains unknown, which was also analyzed in this study.

## Methods

### Study population

This was a retrospective study in the healthy Chinese population who underwent routine health examination carried out between April 1st, 2009 and May 1st, 2017 at Sun Yat-sen Memorial Hospital, Sun Yat-sen University, Guangzhou, China. As described previously [17–19], the population enrolled into this study included the healthy subjects aged 18–89 years old without evidence of acute infection, cardiovascular disease, chronic kidney disease, cancer, diabetes, gout, obstructive pulmonary disease and Alzheimer’s disease based on the previous medical record in Sun Yat-sen Memorial Hospital. Those with various diseases and abnormal medical laboratory tests such as eGFR less than 60 ml/min/1.73m^2^ were excluded from this study. The study protocol was approved by the Institutional Review Boards of the Sun Yat-sen Memorial Hospital and waived the need for informed consent and conducted in accordance with the principles embodied in the Declaration of Helsinki (2013).

### Methods

Serum levels of glucose (Glu), triglyceride (TG), high-density lipoprotein cholesterol (HDL-c), low-density lipoprotein cholesterol (LDL-c), superoxide dismutase (SOD), serum creatinine (SCr), SUA, hs-CRP in fasting blood samples were measured by automated biochemical Analyzer 5800 (BECKMAN) or 7600 (HITACHI). The normal references of serum hs-CRP levels were set at 0–3.0 mg/L. The value of estimated glomerular filtration rate (eGFR) was calculated using the North American Chronic Kidney Disease Epidemiology (CKD-EPI) Collaboration equation.

### Statistical analysis

The baseline characteristics of healthy population and subgroups were analyzed by using SPSS version 19 (IBM Corporation, Armonk, New York, USA). For continuous variables with symmetric distribution, mean data were expressed as mean±standard deviations (*SD*), whereas median and interquartile range (*IQR*) was used for those with asymmetric distribution and examined by Student’s t-test, analysis of variance (ANOVA), chi-square test. Factors found to be statistically significant from univariate analysis (*P* < 0.05) were then tested in multivariate analysis using Linear regression analysis modeling. The correlations between hs-CRP and other risk factors were shown in the scatter plots with linear regression line and *P*-value of < 0.05 from two-sided tests was considered statistically significant.

## Results

### Baseline characteristics of healthy population

**Fig. 1:**
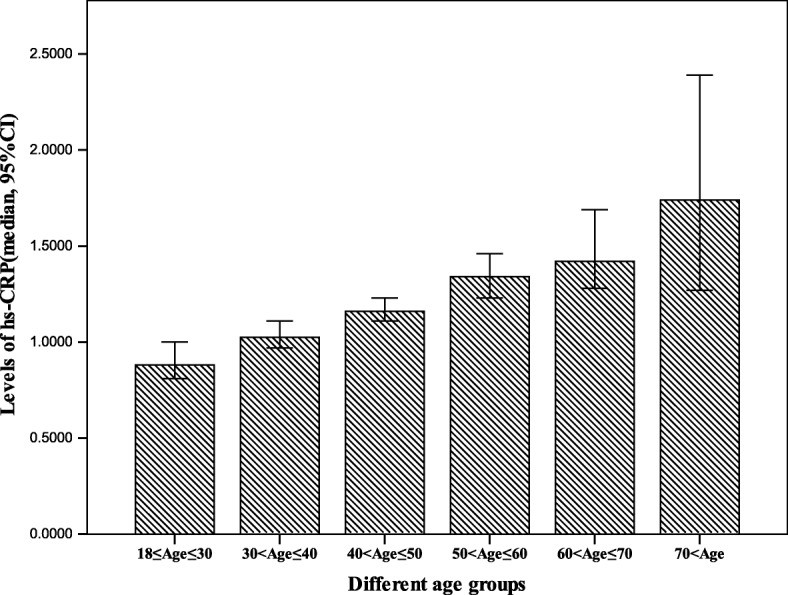
hs-CRP levels in different age groups. Each bar represents the median and 95%CI as indicated. *p < 0.05, ***p < 0.001 compared to the age subgroup of 18–30 year-old population

A total of 6060 healthy people who underwent routine health check were included in this study. Of them, 3672 cases (60.4%) were male and 2388 (39.4%) were female, and the age ranged from 18 to 89 years old with the mean age of45-year-old. Since age of 45 years old is known to be one of the risks for the development of atherosclerotic cardiovascular diseases in men and is the mean menopause age in women with declining steroid hormones and increasing pro-inflammatory markers [3], therefore the age of 45-year-old was stratified as a subgroup for the risk factor of hs-CRP. As shown in Fig. 1, serum levels of hs-CRP were increased with ageing. Compared to male, female population exhibited higher levels of HDL-c and eGFR (*P* < 0.05) but lower levels of hs-CRP, TG, CHOL, LDL-c, SOD, Glu and SUA (*P* < 0.05).The population with age > 45-year-old had higher levels of hs-CRP, TG, CHOL, LDL-c and Glu (*P* < 0.05), but lower levels of eGFR and SOD (*P* < 0.05) compared to age between 18 and 45. There were no differences in levels of SUA and HDL-c (*P* > 0.05) between these two groups (Table 1).

**Table 1:** Baseline characteristics of healthy population

	Overall (*n* = 6060)	Sex subgroup			Age subgroup		
		Male (*n* = 3672)	Female (*n* = 2388)	*P*	18 ≤ Age ≤ 45 (*n* = 3046)	45 < Age (*n* = 3014)	*P*
Age, y	45.90 ± 11.16	45.40 ± 10.97	46.67 ± 11.41	< 0.001	36.98 ± 6.06	54.92 ± 7.18	< 0.001
hs-CRP, mg/L	1.18(0.60, 2.47)	1.24(0.65,2.57)	1.07(0.53,2.29)	< 0.001	1.05(0.53,2.16)	1.31(0.69,2.75)	< 0.001
TG, mmol/L	1.33(0.93, 1.99)	1.53(1.07,2.27)	1.08(0.80,1.57)	< 0.001	1.26(0.87,1.97)	1.39(0.99,2.02)	< 0.001
CHOL, mmol/L	5.35 ± 1.11	5.39 ± 1.13	5.28 ± 1.08	< 0.001	5.19 ± 1.09	5.50 ± 1.11	< 0.001
HDL-c, mmol/L	1.35 ± 0.33	1.26 ± 0.29	1.49 ± 0.34	< 0.001	1.35 ± 0.32	1.36 ± 0.34	0.204
LDL-c,mmol/L	3.35 ± 0.85	3.42 ± 0.85	3.25 ± 0.85	< 0.001	3.24 ± 0.84	3.47 ± 0.85	< 0.001
SOD, U/mL	158.67 ± 32.55	161.33 ± 36.06	154.57 ± 25.72	< 0.001	162.81 ± 21.05	154.48 ± 40.59	< 0.001
eGFR,ml/min.1.73m^2^	83.89 ± 15.04	82.50 ± 14.50	86.02 ± 15.60	< 0.001	88.55 ± 15.77	79.18 ± 12.63	< 0.001
SUA, μmol/L	369.00(307.00,436.75)	409.00[358.00,466.00]	305.00[263.00,353.00]	< 0.001	370.00[303.00,444.00]	367.50(310.00,429.00)	0.184
Glu, mmol/L	5.50 ± 1.58	5.65 ± 1.82	5.28 ± 1.09	< 0.001	5.26 ± 1.31	5.74 ± 1.78	< 0.001

*hs-CRP* High sensitive C-reactive protein, *TG* Triglyceride, *HDL-c* High-density lipoprotein cholesterol, *LDL-c* Low-density lipoprotein cholesterol, *SOD* Superoxide dismutase, *eGFR* Estimated glomerular filtration rate, *SUA* Serum uric acid, *Glu* Glucose

### Risk factors of hs-CRP in the overall healthy population

As shown in Table 2, the univariate linear regression analysis showed that age and levels of SUA and Glu were positively correlated with levels of serum hs-CRP (*P* < 0.05), whereas, serum levels of HDL-c and SOD were negatively correlated with levels of serum hs-CRP (*P* < 0.05). The multivariate linear regression analysis further identified significant correlations between hs-CRP and age (*β* = 0.033, *P* = 0.012), the levels of HDL-c (*β* = − 0.062, *P* < 0.001), SOD (*β* = − 0.100, *P* < 0.001) and SUA (*β* = 0.033, *P* = 0.015).

**Table 2:** The relationship between hs-CRP and biomarkers in healthy population (*N* = 6060)

	Linear regression analysis			
	Univariate analysis		Multivariate analysis	
	*β*	*P*	*β*	*P*
Age	0.046	< 0.001	0.033	0.012
TG	0.005	0.698		
CHOL	−0.004	0.729		
LDL-c	0.002	0.901		
HDL-c	−0.061	< 0.001	−0.062	< 0.001
SOD	−0.096	< 0.001	−0.100	< 0.001
eGFR	−0.007	0.576		
SUA	0.045	< 0.001	0.033	0.015
Glu	0.027	0.034		

### Risk factors of hs-CRP in healthy population stratified by gender

**Fig. 2:**
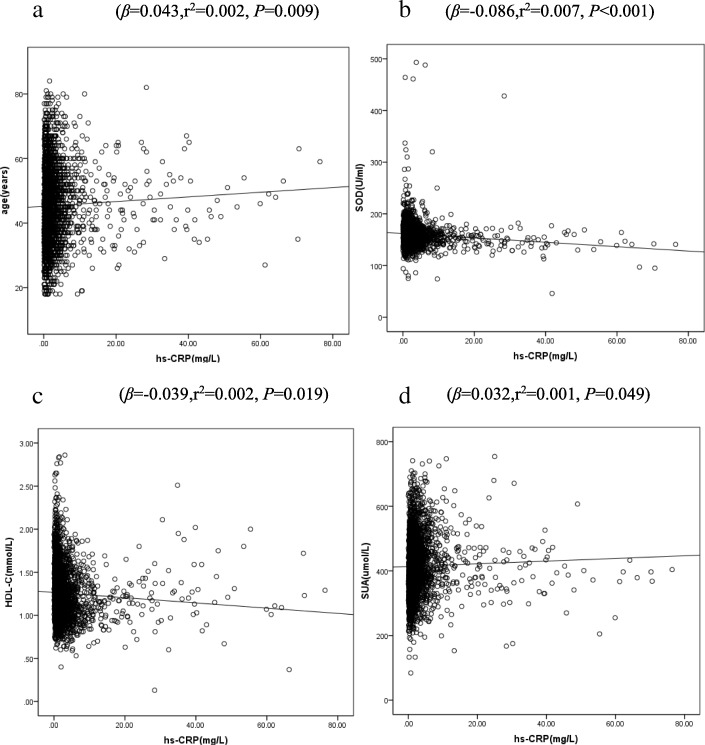
Scatter plot with linear regression line showing the correlations between hs-CRP and age (**a**), SOD (**b**), HDL-C (**c**) and SUA (**d**) in male

**Fig. 3:**
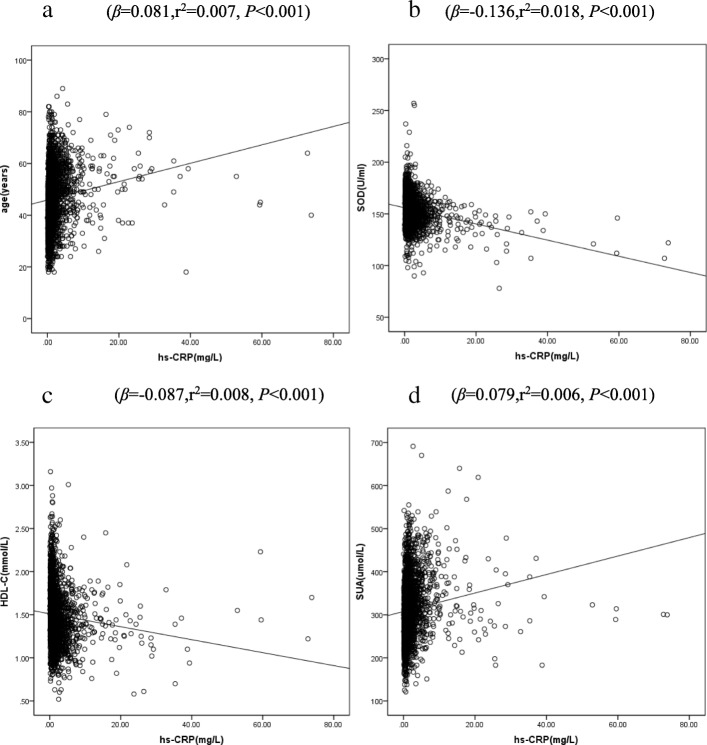
Scatter plot with linear regression line showing the correlations between hs-CRP and age (**a**), SOD (**b**), HDL-C (**c**) and SUA (**d**) in female

Considering the possible effects of gender on serum hs-CRP levels, we analyzed the risk factor of hs-CRP in male and female respectively. In the male subgroup, the multivariate linear regression analysis showed that serum levels of hs-CRP correlated significantly with the age (*β* = 0.037, *P* = 0.026) and SUA (*β* = 0.034, *P* = 0.042), but negatively correlated with the levels of HDL-c (*β* = − 0.041, *P* = 0.014) and SOD (*β* = − 0.086, *P* < 0.001) (Additional file 1: Table S1).The correlations between hs-CRP and these factors in the male population were shown in Figure 2.Similarly, in the female subgroup, results from the multivariate linear regression analysis were generally consistent with the male subgroup (Additional file 1: Table S1).Figure 3 showed the correlations between hs-CRP and these factors in the female population.

### Risk factors of hs-CRP in healthy population stratified by age and gender

A higher level of hs-CRP was associated with ageing and male in 6060 healthy population (Table 1), further analysis was performed to identify the co-risk factors for hs-CRP in healthy people stratified by the mean age and gender. In healthy population with age of less than 45-year-old, the univariate linear regression analysis showed that levels of hs-CRP positively correlated with SUA(*P* < 0.05), but negatively with levels of HDL-c and SOD (*P* < 0.05) in both genders, which were further confirmed by the multivariate linear regression analysis (Tables 3 and 4). Similar results were also found in those with age more than 45-year-old subpopulation (Tables 3 and 4).

**Table 3:** The relationship between hs-CRP and biomarkers in healthy male population

	Linear regression analysis(18 ≤ age ≤ 45) (*n* = 1945)				Linear regression analysis(age > 45)(*n* = 1727)			
	Univariate analysis		Multivariate analysis		Univariate analysis		Multivariate analysis	
	*β*	*P*	*β*	*P*	*β*	*P*	*β*	*P*
Age	0.004	0.846			0.023	0.346		
TG	0.004	0.847			−0.018	0.461		
CHOL	−0.005	0.811			−0.037	0.120		
LDL-C	−0.002	0.935			−0.039	0.108		
HDL-C	−0.064	0.005	−0.060	0.008	−0.081	0.001	−0.081	0.001
SOD	−0.140	< 0.001	−0.143	< 0.001	− 0.065	0.007	− 0.074	0.002
eGFR	−0.012	0.594			0.004	0.860		
UA	0.078	0.001	0.065	0.004	0.055	0.021	0.049	0.041
Glu	0.031	0.177			0.010	0.671

**Table 4:** The relationship between hs-CRP and biomarkers in healthy female population

	Linear regression analysis(18 ≤ age ≤ 45) (*n* = 1101)				Linear regression analysis(age > 45)(*n* = 1287)			
	Univariate analysis		Multivariate analysis		Univariate analysis		Multivariate analysis	
	*β*	*P*	*β*	*P*	*β*	*P*	*β*	*P*
Age	0.016	0.596			0.079	0.005		
TG	0.027	0.374			0.023	0.410		
CHOL	0.034	0.256			−0.002	0.937		
LDL-C	0.041	0.179			0.017	0.547		
HDL-C	−0.110	< 0.001	−0.112	< 0.001	− 0.129	< 0.001	−0.120	< 0.001
SOD	−0.165	< 0.001	−0.169	< 0.001	− 0.139	< 0.001	−0.140	< 0.001
eGFR	0.043	0.154			−0.029	0.297		
UA	0.084	0.005	0.062	0.037	0.142	< 0.001	0.109	< 0.001
Glu	0.006	0.852			0.045	0.103		

## Discussion

The present cross-sectional study revealed that serum levels of hs-CRP were increased with ageing and higher in male than female in Chinese healthy population. Our results showed that higher serum levels of hs-CRP were strongly associated with higher levels of SUA, lower levels of HDL-c and enzymatic antioxidants SOD. These results suggested that elevated serum levels of SUA might trigger CRP production, whereas serum HDL-c and SOD might have an opposite effect. Thus, serum levels of hs-CRP may be a useful indicator for ageing in healthy population.

Ageing is thought to be related to the inflammatory processes. Numerous studies have shown that levels of several cytokines, especially IL-6, TNF alpha and CRP, increase with age in the absence of acute infection [20, 21]. In the present study, we found that elevated levels of hs-CRP levels were correlated with ageing. Interestingly, consistent with previous population-based studies [22, 23], we also found that high serum levels of CRP were significantly correlated with elevated levels of SUA but low levels of HDL-c and SOD. Hyperuricemia has been shown to contribute to the development of hypertension, chronic kidney disease, cardiovascular diseases, type 2 diabetes mellitus and the metabolic syndrome by inducing endothelial dysfunction and pathologic vascular remodeling [24–27]. Thus, SUA and CRP may share common features in vascular remodeling, and it is possible that SUA may trigger CRP expression. Currently, evidence from experimental studies has shown that mitogen-activated protein kinases (MAPK) signaling is a common pathway by which uric acid triggers CRP production in vascular smooth muscle cells and vein endothelial cells [28–30]. It has also been shown that the addition of UA is able to stimulate the expression of CRP by activating the proinflammatory NF-κB signaling cascade [31]. Also, we recently revealed the pathogenic role for CRP in acute and chronic kidney diseases via a NF-κB-dependent mechanism [32–34]. Furthermore, changes of hs-CRP have been applied as a biomarker clinically as evidenced by the lowering of serum CRP in response to various treatments under disease conditions [35–39].Taken together, results from the present study suggested that CRP might not only be a biomarker and mediator for inflammatory disease, but also as a sensitive indicator for the healthy ageing.

The gender difference with respect to the serum levels of CRP has not been fully clarified. Previous studies have shown a higher prevalence of high hs-CRP concentrations in Inuit or Pakistan women populations than that in men [40, 41]. In our study, we found that higher levels of hs-CRP were more prevalent in male than female individuals. This maybe partly attributed to the higher level of SUA but lower level of HDL-c. Previous studies also reported that circulating levels of CRP in elderly subjects were positively correlated with levels of blood glucose and HbA1c, but inversely correlated with renal dysfunction [42–45]. However, in this population-based study, we could not find any relationship between hs-CRP and Glu or eGFR. This may be largely due to the normal levels of Glu and eGFR in the healthy population.

There are some limitations in this study. First, since this study was retrospectively based on the healthy population who underwent routine healthy examination at clinic, we are unable to acquire data regarding BMI, smoking habit, and other information that are potentially associated with the occurrence of inflammation and their consequences [3]. Second, since CRP was co-existing with other inflammatory conditions, our retrospective data may not be able to exclude those with minor acute inflammation, although the enrolled subjects were apparently healthy. Finally, since the majority of our study subjects were limited to those with age between 30 and 60 years old, the study outcomes may be more generalized to young and middle-aged healthy people, despite the fact that high serum levels of CRP were found in the population with age more than 65 years old.

## Conclusions

This retrospective study shows that serum levels of hs-CRP are increased with ageing, particularly in male than female healthy population. Furthermore, elevated serum levels of hs-CRP are associated with high levels of SUA, but low levels of HLD-c and SOD, suggesting that the inflammatory status may also contribute to ageing in the healthy population.

## Additional file

Additional file 1: **Table S1.** The relationship between hs-CRP and biomarkers stratified by gender in healthy population. **Table S2.** The relationship between hs-CRP and biomarkers stratified by age in healthy population. (DOCX 20 kb)

## Abbreviations

ANOVA: Analysis of variance
CHOL: Cholesterol
CRP: C-reactive protein
CVD: Cardiovascular disease
eGFR: Estimated glomerular filtration rate
Glu: Glucose
HDL-c: High-density lipoprotein cholesterol
hs-CRP: High sensitive C-reactive protein
IL-6: Interleukin-6
IQR: Interquartile range
LDL-c: Low-density lipoprotein cholesterol
SCr: Serum creatinine
SD: Standard deviation
SOD: Superoxide dismutase
SUA: Serum uric acid
TG: Triglyceride
