# Supplementary material for: The baseline levels and risk factors for high-sensitive C-reactive protein in Chinese healthy population
Source: Immun Ageing. 2018 Sep 8;15:21. doi: 10.1186/s12979-018-0126-7 (PMC6128988; doi:10.1186/s12979-018-0126-7)
Supplement: Supplementary file 1 — Table S1. The relationship between hs-CRP and biomarkers stratified by gender in healthy population. Table S2. The relationship between hs-CRP and biomarkers stratified by age in healthy population. (DOCX 20 kb) [file 12979_2018_126_MOESM1_ESM.docx]

**Additional file 1**

**Table S1.The relationship between hs-CRP and biomarkers stratified by gender in healthy population**

|  | **Linear regression analysis(male)** | | | | | | | **Linear regression analysis(female)** | |  |
| --- | --- | --- | --- | --- | --- | --- | --- | --- | --- | --- |
|  | **Univariate analysis** | | | **Multivariate analysis** | | | | **Univariate analysis Multivariate analysis** | |  |
|  | ***β*** | ***P*** | | ***β*** | | ***P*** | | ***β P β P*** | |  |
| Age  TG  CHOL | 0.043  -0.009  -0.023 | | 0.009  0.570  0.170 | | 0.037 | | 0.026 | | 0.081 <0.001 0.043 0.043  0.028 0.173  0.028 0.175 |  |
| LDL-c | -0.022 | | 0.182 | |  | |  | 0.039 0.057 | |  |
| HDL-c | -0.039 | | 0.019 | | -0.041 | | 0.014 | -0.087 <0.001 -0.085 <0.001 | |  |
| SOD | -0.086 | | <0.001 | | -0.086 | | <0.001 | -0.136 <0.001 -0.131 <0.001 | |  |
| eGFR | -0.016 | | 0.334 | |  | |  | 0.016 0.430 | |  |
| SUA | 0.032 | | 0.049 | | 0.034 | | 0.042 | 0.079 <0.001 0.044 0.036 | |  |
| Glu | 0.022 | | 0.180 | |  | |  | 0.033 0.110 | |  |

**Table S2.The relationship between hs-CRP and biomarkers stratified by age in healthy population**

|  | **Linear regression analysis (18**≤**Age**≤**45)** | | | | | | | **Linear regression analysis (Age>45)** | |
| --- | --- | --- | --- | --- | --- | --- | --- | --- | --- |
|  | **Univariate analysis** | | | **Multivariate analysis** | | | | **Univariate analysis Multivariate analysis** | |
|  | ***β*** | ***P*** | | ***β*** | | ***P*** | | ***β P β P*** | |
| Age  TG  CHOL | 0.010  0.015  0.015 | | 0.569  0.409  0.399 | |  | |  | | 0.036 0.050  -0.004 0.815  -0.029 0.111 |
| LDL-c | 0.022 | | 0.232 | |  | |  | -0.023 0.203 | |
| HDL-c | -0.046 | | 0.010 | | -0.045 | | 0.019 | -0.074 <0.001 -0.073 <0.001 | |
| SOD | -0.135 | | <0.001 | | -0.145 | | <0.001 | -0.077 <0.001 -0.086 <0.001 | |
| eGFR | 0.021 | | 0.242 | |  | |  | -0.010 0.573 | |
| SUA | 0.056 | | 0.002 | | 0.054 | | 0.005 | 0.053 0.003 0.038 0.046 | |
| Glu | 0.024 | | 0.180 | |  | |  | 0.020 0.266 | |
